# Influence of light exposure at nighttime on sleep development and body growth of preterm infants

**DOI:** 10.1038/srep21680

**Published:** 2016-02-15

**Authors:** Yosuke Kaneshi, Hidenobu Ohta, Keita Morioka, Itaru Hayasaka, Yutaka Uzuki, Takuma Akimoto, Akinori Moriichi, Machiko Nakagawa, Yoshihisa Oishi, Hisanori Wakamatsu, Naoki Honma, Hiroki Suma, Ryuichi Sakashita, Sei-ichi Tsujimura, Shigekazu Higuchi, Miyuki Shimokawara, Kazutoshi Cho, Hisanori Minakami

**Affiliations:** 1 Maternity and Perinatal Care Center, Hokkaido University Hospital, N15, W7, Kita-ku, Sapporo 060-8638, Japan; 2 Department of Developmental Disorders, National Institute of Mental Health, National Center of Neurology and Psychiatry, 4-1-1 Ogawa-higashi-machi, Kodaira, Tokyo 187-8553, Japan; 3 Department of Pediatrics, St. Luke’s International Hospital, 9-1 Akashi-cho, Chuo-ku, Tokyo 104-8560, Japan; 4 Department of Pediatrics, Japanese Red Cross Medical Center, 4-1-22 Hiroo, Shibuya-ku, Tokyo 150-8935, Japan; 5 Atom Medical Corp., 2-2-1 Michiba, Sakura-ku, Saitama 338-0835, Japan; 6 Luceo Co., Ltd., 30-9 Ohyamakanai-cho, Itabashi-ku, Tokyo 173-0024, Japan; 7 Faculty of Sciences and Engineering, Kagoshima University, 1-21-40, Koorimoto, Kagoshima 890-0065, Japan; 8 Department of Human Science, Faculty of Design, Kyushu University, 9-1, Shiobaru 4-chome, Minami-ku, Fukuoka 815-8540, Japan; 9 Department of Obstetrics, Hokkaido University Graduate School of Medicine, N15, W7, Kitaku, Sapporo 060-8638, Japan

## Abstract

Previous studies have demonstrated that a light-dark cycle has promoted better sleep development and weight gain in preterm infants than constant light or constant darkness. However, it was unknown whether brief light exposure at night for medical treatment and nursing care would compromise the benefits brought about by such a light-dark cycle. To examine such possibility, we developed a special red LED light with a wavelength of >675 nm which preterm infants cannot perceive. Preterm infants born at <36 weeks’ gestational age were randomly assigned for periodic exposure to either white or red LED light at night in a light-dark cycle after transfer from the Neonatal Intensive Care Unit to the Growing Care Unit, used for supporting infants as they mature. Activity, nighttime crying and body weight were continuously monitored from enrolment until discharge. No significant difference in rest-activity patterns, nighttime crying, or weight gain was observed between control and experimental groups. The data indicate that nursing care conducted at 3 to 4-hour intervals exposing infants to light for <15 minutes does not prevent the infants from developing circadian rest-activity patterns, or proper body growth as long as the infants are exposed to regular light-dark cycles.

Circadian rhythms are endogenously generated rhythms that have a period length of about 24 hours^1^. Evidence gathered over the past decade indicates that the circadian clock in the suprachiasmatic nuclei (SCN) in the anterior hypothalamus develops prenatally by mid-gestation in non-human primates and humans^2^,^3^. Human preterm infants can respond to light, possibly through the retinal ganglion cells containing melanopsin in the retina, as early as the corrected gestational age of 30 weeks and start to develop circadian behavioral rhythms in a light-dark (LD) cycle^4^,^5^,^6^,^7^. The rod cells containing rhodopsin in the retina also seem to respond to light at around 34 weeks’ gestational age (WGA)^8^,^9^. In full-term infants, circadian system outputs mature progressively after birth, with rhythms in body temperature, sleep-wake cycles, and hormone production generally developing between 0 and 3 months of age^10^.

The importance of regular LD cycles in early human development is demonstrated by the fact that the establishment of circadian rest-activity patterns and postnatal weight gains among preterm infants is accelerated in Neonatal Intensive Care Units (NICUs) that employ regular LD cycles^6^,^8^,^10^,^11^,^12^. In contrast, continuous light or dark conditions delay the onset of circadian behavioral rhythms and lead to reduced weight gain in preterm infants^8^,^11^,^12^. In addition, pregnant women exposed to irregular LD cycles have been reported to have increased rates of reproductive abnormalities such as miscarriage, preterm delivery, and low birth weight of offspring^13^,^14^. A recent animal study also demonstrated that the exposure of pregnant rats to constant light, which disrupts the circadian environment of fetuses, induces fetal growth retardation *in utero*^15^.

Even among preterm infants and neonates raised in regular LD cycles, there is still a possibility that light exposure during nighttime for medical and nursing care disrupts their development of circadian systems. In adults, nighttime exposure to white light or light-emitting eBooks has been reported to affect their circadian rhythms^16^,^17^,^18^,^19^,^20^,^21^,^22^. Preterm infants in NICUs also experience repeated 15–30-min exposures of white LED light every 2–4 hours during feeding and diaper changes. To protect preterm infants from the possible negative effects of nighttime lighting on their development in the NICU, we have developed a red LED light with a wavelength of >675nm, which preterm infants cannot detect due to the prematurity of their retinal photoreceptors such as cone cells, which mature at a later developmental stage^4^,^7^,^8^,^9^. In this study, we investigated whether nighttime nursing care using conventional white LED light has some adverse effects on establishment of circadian rhythm and weight gain in preterm infants compared to nursing care conducted using our developed red LED light.

## Results

### Characteristics of Study Infants

A total of 42 infants were enrolled in this study. The characteristics of these infants are shown in Table 1 (mean ± s.d., throughout). Between the control and experimental groups, no statistical differences were observed in birth weight, gestational age at birth, gestational age at enrollment, length of intervention, discharge weight, discharge head circumference, or maternal age (Table 1, t-test; P > 0.05).

### Lighting Conditions

**Figure 1:**
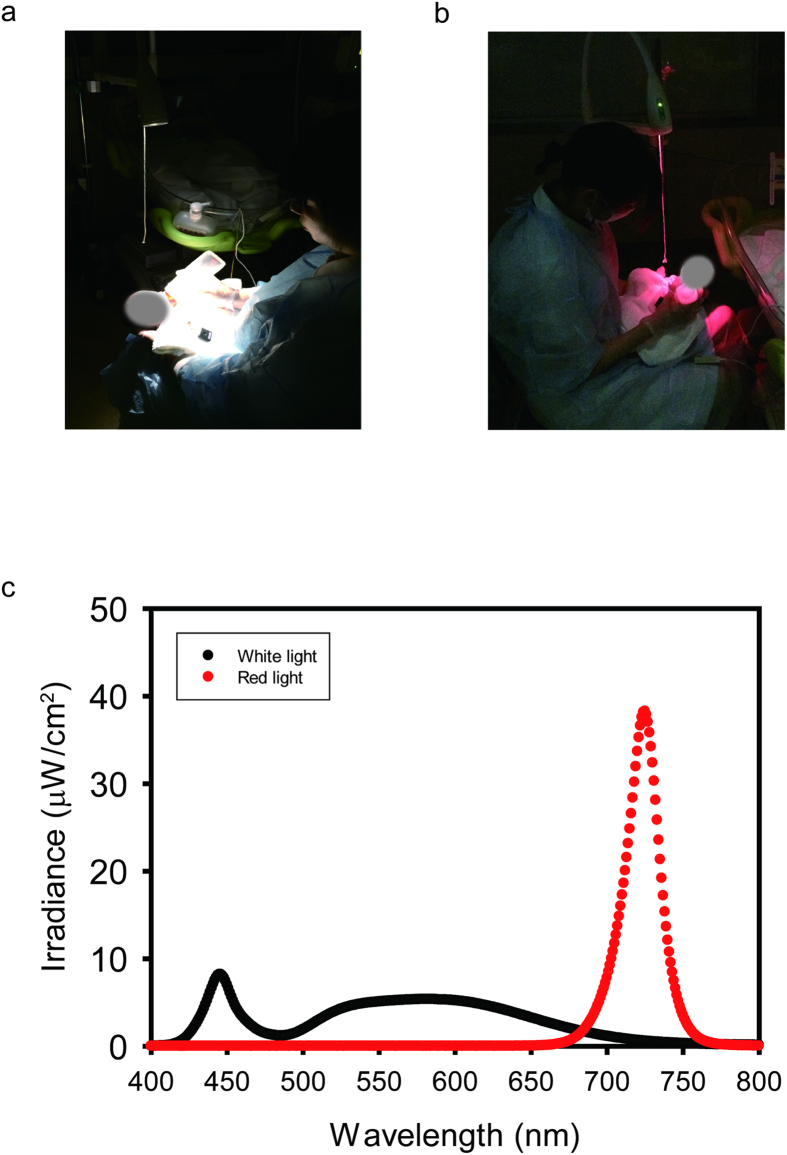
Relative spectral distribution of a white LED light and a red LED light. Representative pictures of nursing care under white LED light (**a**) and red LED light (**b**). (**c**) White line indicates relative spectral distribution of white LED light while red line indicates relative spectral distribution of red LED light. Red LED light has a peak emission of approximately 725 nm.

In both the control (white LED light, Fig. 1a) and experimental groups (red LED light, Fig. 1b), daytime lighting exposure from 06:00 to 21:00 h was 44.4 ± 10.7 and 45.7 ± 14.1 lux, respectively; there was no statistical difference in background irradiance between the two groups during the daytime (t-test; P > 0.05).

### Activity Assessment

**Figure 2:**
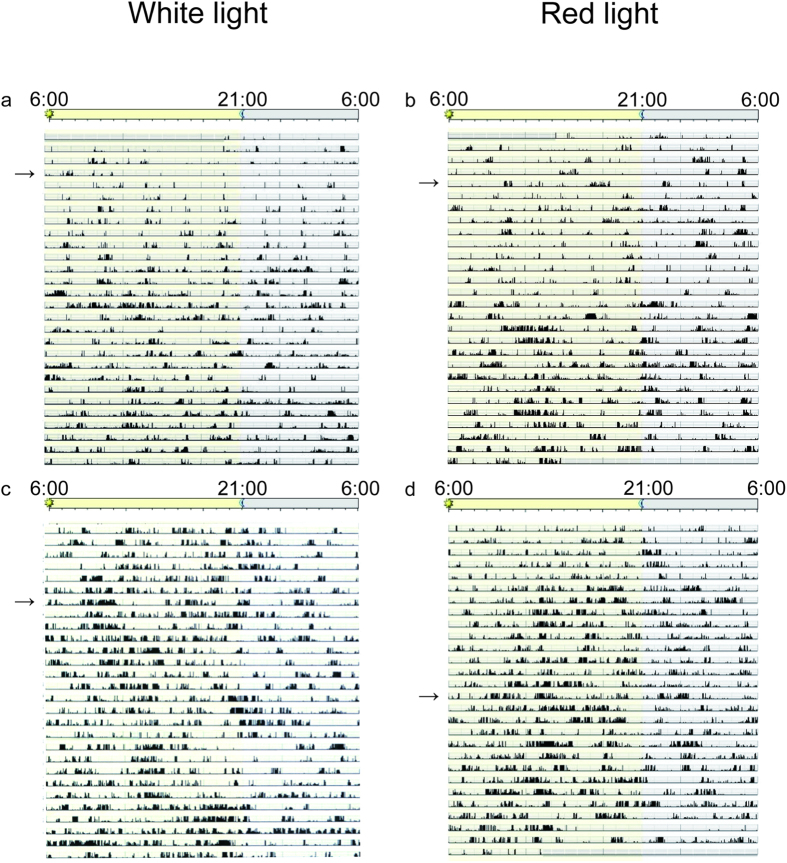
Actograms of rest-activity in representative infants who were exposed to white LED light (**a**,**c**) or red LED light (**b**,**d**) during night in an LD cycle. Dark bars represent activity; the same activity scale is used in each plot. The time of day is shown at the top. Actograms from a representative infant exposed to either white LED light (**a**) or red LED light (**b**) at around 35 WGA. Activity bouts occur every 2–3 hours associated with nursing care such as feeding and diaper changes. The arrows in the plots depict the date of 35 weeks of gestational age (WGA). Actograms from a representative infant exposed to either white LED light (**c**) or red LED light (**d**) at around 38 WGA. The arrows in the plots depict the date of 38 WGA. Note that more active patterns during daytime in both groups of infants are apparent after around 38 WGA in both groups of infants regardless of the wavelength of LED light used.

Actograms were generated for each infant from the time of enrollment at around 35 weeks’ gestational age (WGA) until discharge from the hospital at around 40 WGA (Fig. 2). Actogram data were first analyzed to assess whether there were differences among the groups in the absolute numbers of movements per hour. No differences between control and experimental group infants during both daytime and nighttime were observed for each of the 7-day periods examined. (Table 2, t-test; P > 0.05).

**Figure 3:**
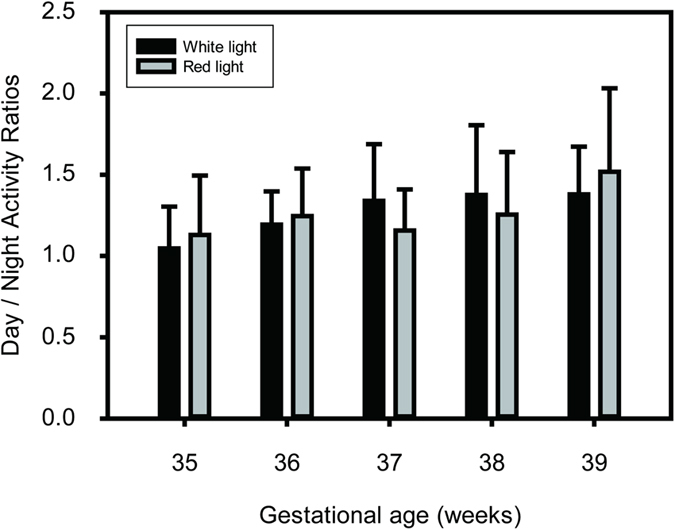
Day-night activity ratios over consecutive 7-day periods, at 35, 36, 37, 38, and 39 weeks of gestational age (WGA). Dark bars: control infants exposed to white LED light during night; gray bars: experimental group infants exposed to red LED light during night (mean ± s.d.). Observe that exposure to either white or red LED light results in similar patterns of increased daytime activity over nighttime activity over each range of days.

Next, actogram data were examined to assess whether the distribution of activity varied between day (06:00–21:00 h) and night (21:00–06:00 h) (Fig. 3). When actograms of both the control and experimental infants were examined, slight day-night differences in rest and activity were already observed in most infants at 35 WGA. Ratios of day-night activity were 1.06 ± 0.13 for control infants, indicating 6% more total activity during the day than at night. When actograms of experimental group infants were inspected at 35 WGA, the day-night activity ratio was 1.24 ± 0.47, indicating 24% more activity during the day than at night.

To evaluate differences in day-night activity ratios between the two groups over time, two-way repeated measures ANOVA was performed with lighting conditions (white or red light) as the between-group variables and gestational age (35–39 WGA) as the within-group variables. There were no statistically significant differences between the white and red light groups (F (1, 142) = 0.193; P > 0.05), nor was there a significant interaction of groups x age (F (4, 142) = 0.809; P > 0.05), indicating that the day-night activity ratios were not affected by type of light. There was a significant effect of gestational age (F(4, 142) = 2.597; P < 0.05), indicating more daytime activity than nighttime activity as the infants became older.

### Crying Assessment

**Figure 4:**
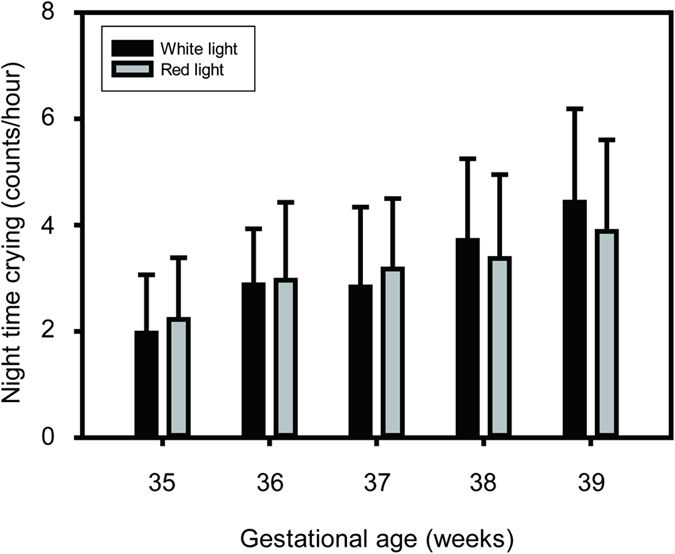
Nighttime crying over consecutive 7-day periods, at 35, 36, 37, 38, and 39 weeks of gestational age (WGA). Dark bars: control infants exposed to white LED light during night; gray bars: experimental group infants exposed to red LED light during night (mean ± s.d.). Observe that exposure to white or red LED lighting results in similar night crying counts over each range of days.

Nighttime crying episodes were counted for each infant from the time of enrollment at around 35 WGA until discharge from the hospital at around 40 WGA (Fig. 4). To evaluate differences in nighttime crying counts between the groups over time, two-way repeated measures ANOVA was performed as above. There were no significant effects of lighting condition (F (1, 146) = 0.143; P > 0.05) or groups x age interaction (F(4, 146) = 0.797; P > 0.05), indicating that the nighttime crying counts were not affected by type of light. There was a significant increase in crying bouts as the infants became older (F(4, 146) = 6.449; P < 0.01).

### Growth

**Figure 5:**
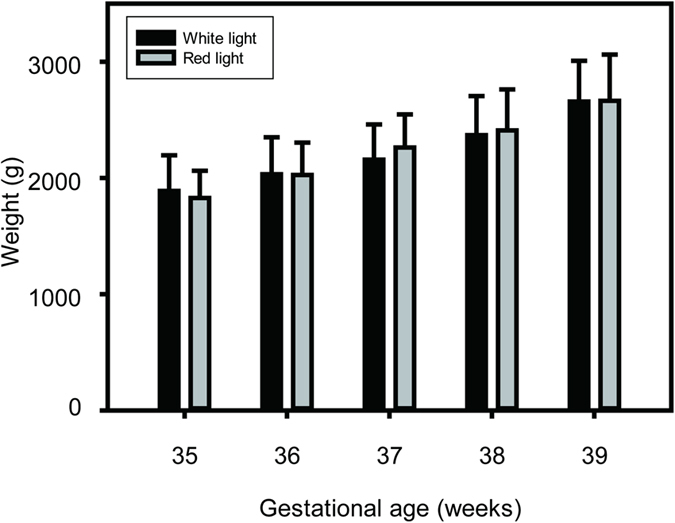
Body weight gains over consecutive 7-day periods, at 35, 36, 37, 38, and 39 weeks of gestational age (WGA). Dark bars: control infants exposed to white LED light during night; gray bars: experimental group infants exposed to red LED light during night (mean ± s.d.). Observe that exposure to white or red LED lighting results in similar weight gains over each range of days.

Body weight was measured daily for each infant from the time of enrollment at around 35 WGA until discharge from the hospital at around 40 WGA (Fig. 5). Repeated measures ANOVA was used to assess the effects of lighting condition and gestational age on body growth. There were no significant differences between the white and red light groups (F (1, 138) = 0.099; P > 0.05) and in the interaction of groups x age (F (4, 138) = 0.261; P > 0.05), indicating that the body weights were not affected by type of light. There was a significant increase in body weights as the infants became older (F(4, 138) = 24.965; P < 0.01).

## Discussion

This study is the first to examine the effects of brief light pulses during nighttime on the developing circadian clocks of preterm infants in an light-dark cycle. We employed a white LED light for use with the control group and developed a red LED light for use with the experimental group. The red LED light has a wavelength of >675 nm, which preterm infants cannot detect due to the prematurity of their retinal photoreceptors such as cone cells, which mature at a later developmental stage^4^,^7^,^8^,^9^. The red LED device produces light with a peak wavelength of 725 nm and a half value width of 50 nm, which is outside the range of perception of infants, while the white LED device produces light made up of a broad range of wavelengths including those that infants can perceive. We hypothesized that exposure to white LED light disrupts the development of circadian rest-activity patterns leading to more nighttime crying and less weight gain, compared to exposure to red LED light. The present study, however, demonstrated that there was no significant difference in rest-activity patterns, nighttime crying, or weight gains between preterm infants exposed to normal white LED light (3480 lux) and those exposed to red LED light (21 lux) while receiving regular repeated nursing care during night in a GCU, which maintained a daily 15-hour: 9-hour light-dark cycle.

In human adults in constant darkness, the circadian clock has been reported to be responsive to brief light pulses in the evening. A single 2-hour bright light pulse of approximately 4000 lux in the late evening phase delayed the circadian rhythm of melatonin an average of approximately 1.5 hours^19^. As for EEG, a single 2-hour blue light pulse (460 nm) in the evening affects REM sleep and sleep EEG power density of slow-wave activity^18^,^21^. Moreover, 3–5 consecutive days of exposure to intermittent pulses of bright light (3000–11000 lux) as brief as 5 min in duration in the evening have been shown to induce 2.3-hour phase advances of the circadian rhythm of melatonin^17^.

In studies of nocturnal animals in constant darkness also, it has been reported that the mammalian circadian clock is responsive to brief exposures of light. In rodents such as mice, rats and hamsters, brief bright light pulses from 10 μs to 5 min in constant darkness have been reported to phase shift their circadian activity rhythms^23^,^24^,^25^. A single 30 min light pulse of 1000 lux in the late evening in constant darkness affects the expression of a clock gene, Period1, and/or circadian activity rhythms in mice and rats^26^,^27^. Since the pupillary light reflex of mice has a much lower threshold than humans and is sensitive to a more divergent range of wavelengths, lower levels and shorter durations of light exposure have been reported to be able to affect mouse circadian systems^28^.

One possible explanation for the absence of effects of white LED light on infants’ physiology could be that the effects of regular light-dark cycles overwhelm those from brief white LED light exposure at night. In most previous studies of both humans and animals, the phase shifts induced by a brief light exposure in the evening were observed not in light-dark cycles, but in constant darkness or constant dim light of <10 lux. In constant darkness or dim light, a larger phase shift can be induced than in subjects exposed to regular light-dark cycles^22^,^29^. In the present study, the larger resetting effects of light-dark cycles may have erased the possible phase shifts in preterms’ activity rhythms induced by a brief light exposure in the evening.

Another possible explanation for the absence of effects of red LED light on infants’ physiology could be that appropriate parameters to assess the effects of red LED light on infants have not been selected. For instance, it may be that white LED light, but not red LED light, stimulated cortisol secretion and suppressed melatonin in these infants without alerting the measured outcome variables. However, since non-invasive methods such as measurement of saliva cortisol, electroencephalography (EEG), and electrocardiogram (ECG) do not show stable circadian rhythmicy at around term, assessment of these cannot be utilized to assess the circadian development of the preterm infants in this study^30^,^31^,^32^,^33^. There is evidence in rats that only short wave lengths (blue) affect hormone secretion: in one study, selectively filtering blue light into short wavelengths between 452 and 462 nm prevented the rise of corticosterone and restored normal melatonin secretion as well as restored clock gene expression patterns in the hypothalamus and adrenal glands^34^. Further studies to examine wavelength effects on infant hormone secretion could be of interest, but would be ethically difficult to perform since they would require blood sampling.

In clinical practice the red LED light had a relatively low illuminance level of approximately 21 lux (compared to the 3480 lux generated by the white LED light), but was bright enough for the staff to perform necessary nursing care such as feeding and diaper changes. The use of the red LED light also made it more difficult for the staff to check for apnea in infants from their skin color. However, in practice the red LED light did not prevent the staff from being able to check for apnea in the infants since oxygen saturation monitoring systems are routinely attached to all the infants in the Growing Care Unit (GCU).

In summary, this study confirmed no significant difference in rest-activity patterns, nighttime crying or weight gains between preterm infants exposed to normal white LED light and those exposed to red LED light for regular nursing care during the night at a GCU in a 15-hour: 9-hour light-dark cycle. Furthermore, this study also demonstrated that exposing infants to light for <15 minutes at 3 to 4 hour intervals for nursing care does not prevent the infants from developing normal circadian rest-activity patterns or gaining weight as long as the infants are exposed to regular light-dark cycles.

## Methods

### Participants

Forty-two preterm infants who were born at <36 weeks’ gestational age (WGA) and hospitalized at Hokkaido University Hospital were studied in an LD (15 h light: 9 h dark) cycle. After transfer from the NICU to the Growing Care Unit (GCU), used for supporting infants as they mature, medically stable infants were randomly assigned to either white (control group, n = 21) or red (experimental group, n = 21) LED light exposure during nighttime nursing care, using a table of random numbers (Table 1). Infants were eligible for enrollment if they had no current significant medical problems, had no significant eye disease (retinopathy of prematurity grades 3 or 4), had no major congenital malformations, and had no major neurological disorders (including intraventricular hemorrhage grades 3 or 4 or periventricular leukomalacia). Infants were also excluded if they had had intensive treatments for more than one week because of medical complications such as bacterial infection. The ethics committee of Hokkaido University Hospital approved this study protocol (UMIN000014859) and all procedures were carried out in accordance with the approved guidelines. Written informed consent was obtained from the parents.

### Lighting conditions and monitoring of light intensity

Background lighting in the GCU was provided by overhead LED lighting of 40–100 lux during a daytime of 06:00–21:00 h and 1–15 lux during a nighttime of 21:00–06:00 h. The light intensity of background lighting was continuously monitored using an Actiwatch-L light sensor (Minimitter Co., OR, USA), which was placed in each isolette near the head of the infant during hospitalization.

During nighttime, nursing care such as feeding and diaper changes were provided approximately every three hours. During nursing care, the control group (white LED light group) was exposed to a white LED light which produced approximately 1100 μW/cm^2^ at a distance of 40 cm (Fig. 1a, Atom Medical Co., Saitama, Japan). In the experimental group (red LED light group), a red LED light was used for the same care procedures as in the control group (Fig. 1b, peak wavelength of 725 nm; 1100 μW/cm^2^ at 40 cm). 1100 μW/cm^2^ was set for both white and red light groups so that the same light power density reached the eyes of both groups of infants. The illuminance levels for the white and red LED lights for human adults were 3480 lux and 21 lux, respectively (T-10, Konica Minorta Corp., Tokyo, Japan). Both the white and red LED light were bright enough for the medical staff to perform nursing care such as feeding and diaper changes at night. Relative spectral distributions of two lights are shown in Fig. 1c.

### Activity Assessment

Infant activity was monitored continuously using an Actiwatch-L (Minimitter Co., OR, USA), which was also used as a light sensor. An Actiwatch-L was attached on one ankle of each infant using a soft sleeve bandage. The Actiwatch-L records a digitally integrated measure of gross motor activity that is assessed by an internal accelerometer, yielding an absolute number of movements per hour (watch sensitivity was <0.01 N). Daytime and nighttime activity levels were defined by the numbers of movements per hour during the intervals of 06:00–21:00 h and 21:00–06:00 h, respectively. For assessing whether there was diurnal variation in movement, day-night activity ratios were calculated by dividing the daytime activity level by the nighttime activity level^6^,^35^. A day-night activity ratio greater than 1.0 indicates that the infant is more active during daytime than nighttime.

### Crying Assessment

Nurses recorded each crying episode in the infant’s medical chart when they aurally recognized crying during the nighttime. The number of crying episodes during the nighttime was collected from these medical charts.

### Growth

Infants were weighed daily at 09:00–10:00 h using a calibrated scale. Daily weight gain for each week of the infant’s hospitalization was calculated as the average of 7 consecutive daily weight gains.

### Statistical Analysis

Sample size was determined by power analysis using Sample-Power Version 1.0 (SPSS, Inc, Chicago, IL). For a 2-tailed α level of 0.05, we calculated that 21 subjects in each group would be needed to observe a large effect size of 1.0 with a power of 0.90. Student’s t-test and two-way repeated measures ANOVA were used for group comparisons using SPSS Version 20.0 (SPSS, Inc, Chicago, USA).

## Additional Information

**How to cite this article**: Kaneshi, Y. *et al*. Influence of light exposure at nighttime on sleep development and body growth of preterm infants. *Sci. Rep.* **6**, 21680; doi: 10.1038/srep21680 (2016).

## Figures and Tables

**Table 1:** Patient characteristics.

	White light group (*n* = 21)	Red light group (*n* = 21)
Birth weight, g	1532 ± 465	1568 ± 375
Gestational age at birth, week	32.3 ± 2.2	32.4 ± 1.8
Gestational age at enrollment, week	36.1 ± 1.3	35.8 ± 1.8
Length of intervention, day	29.9 ± 10.8	33.7 ± 12.6
Discharge weight, g	2907 ± 485	2967 ± 631
Discharge head circumference, cm	35.0 ± 1.7	34.6 ± 1.5
Maternal age	35.2 ± 4.5	34.0 ± 4.3

All comparisons between groups (mean ± s.d., t-test; *P* > *0*.05).

**Table 2:** Daytime and nighttime activity counts.

Gestational weeks of age (WGA)	Daytime (counts/hour)		Nighttime (counts/hour)	
	White light	Red light	White light	Red light
35	3245 ± 1840	2792 ± 824	3346 ± 1638	2823 ± 1119
36	3693 ± 1499	3466 ± 1323	3358 ± 1202	3179 ± 1440
37	4222 ± 1523	3904 ± 1464	3813 ± 1405	3714 ± 1277
38	4604 ± 1393	4178 ± 1334	4019 ± 1391	3774 ± 1454
39	4735 ± 1062	4810 ± 1422	4087 ± 1429	3783 ± 1273

All comparisons between white and red light groups (mean ± s.d., t-test; *P* > *0*.05).

## References

[b1] Reppert S. M. & Weaver D. R. Coordination of circadian timing in mammals. Nature 418, 935–941 (2002). 12198538 10.1038/nature00965

[b2] Reppert S. M., Weaver D. R., Rivkees S. A. & Stopa E. G. Putative melatonin receptors in a human biological clock. Science 242, 78–81 (1988). 2845576 10.1126/science.2845576

[b3] Serón-Ferré M. . Perinatal neuroendocrine regulation. Development of the circadian time-keeping system. Mol Cell Endocrinol 186, 169–173 (2002). 11900892 10.1016/s0303-7207(01)00682-7

[b4] Robinson J. & Fielder A. R. Pupillary diameter and reaction to light in preterm neonates. Arch Dis Child 65, 35–38 (1990). 2306132 10.1136/adc.65.1_spec_no.35 PMC1590160

[b5] Hao H. & Rivkees S. A. The biological clock of very premature primate infants is responsive to light. Proc Natl Acad Sci USA 96, 2426–2429 (1999). 10051658 10.1073/pnas.96.5.2426 PMC26800

[b6] Rivkees S. A., Mayes L., Jacobs H. & Gross I. Rest-activity patterns of premature infants are regulated by cycled lighting. Pediatrics 113, 833–839 (2004). 15060235 10.1542/peds.113.4.833

[b7] Hanita T., Ohta H., Matsuda T. & Miyazawa H. Monitoring preterm infants’ vision development with light-only melanopsin is functional. J Pediatr 155, 596–596.e591 (2009). 19773007 10.1016/j.jpeds.2009.03.005

[b8] Watanabe S. . Designing Artificial Environments for Preterm Infants Based on Circadian Studies on Pregnant Uterus. Front Endocrinol (Lausanne) 4, 113 (2013). 24027556 10.3389/fendo.2013.00113 PMC3761559

[b9] Ikeda T., Ishikawa H., Shimizu K., Asakawa K. & Goseki T. Pupillary size and light reflex in premature infants. Neuro-Ophthalmol 39, 175–178 (2015). 10.3109/01658107.2015.1055363 PMC5123060 27928351

[b10] Rivkees S. A. Developing circadian rhythmicity in infants. Pediatr Endocrinol Rev 1, 38–45 (2003). 16437011

[b11] Morag I. & Ohlsson A. Cycled light in the intensive care unit for preterm and low birth weight infants. Cochrane Database Syst Rev 8, CD006982 (2013). 23913547 10.1002/14651858.CD006982.pub3

[b12] Vásquez-Ruiz S. . A light/dark cycle in the NICU accelerates body weight gain and shortens time to discharge in preterm infants. Early Hum Dev 90, 535–540 (2014). 24831970 10.1016/j.earlhumdev.2014.04.015

[b13] Cone J. E., Vaughan L. M., Huete A. & Samuels S. J. Reproductive health outcomes among female flight attendants: an exploratory study. J Occup Environ Med 40, 210–216 (1998). 9531091 10.1097/00043764-199803000-00002

[b14] Aspholm R. . Spontaneous abortions among Finnish flight attendants. J Occup Environ Med 41, 486–491 (1999). 10390700 10.1097/00043764-199906000-00015

[b15] Mendez N. . Timed maternal melatonin treatment reverses circadian disruption of the fetal adrenal clock imposed by exposure to constant light. PLoS One 7, e42713 (2012). 22912724 10.1371/journal.pone.0042713 PMC3418288

[b16] Khalsa S. B., Jewett M. E., Cajochen C. & Czeisler C. A. A phase response curve to single bright light pulses in human subjects. J Physiol 549, 945–952 (2003). 12717008 10.1113/jphysiol.2003.040477 PMC2342968

[b17] Gronfier C., Wright K. P., Kronauer R. E., Jewett M. E. & Czeisler C. A. Efficacy of a single sequence of intermittent bright light pulses for delaying circadian phase in humans. Am J Physiol Endocrinol Metab 287, E174–181 (2004). 15039146 10.1152/ajpendo.00385.2003 PMC2761596

[b18] Münch M. . Wavelength-dependent effects of evening light exposure on sleep architecture and sleep EEG power density in men. Am J Physiol Regul Integr Comp Physiol 290, R1421–1428 (2006). 16439671 10.1152/ajpregu.00478.2005

[b19] Canton J. L., Smith M. R., Choi H. S. & Eastman C. I. Phase delaying the human circadian clock with a single light pulse and moderate delay of the sleep/dark episode: no influence of iris color. J Circadian Rhythms 7, 8 (2009). 19615064 10.1186/1740-3391-7-8 PMC2722576

[b20] St Hilaire M. A. . Human phase response curve to a 1 h pulse of bright white light. J Physiol 590, 3035–3045 (2012). 22547633 10.1113/jphysiol.2012.227892 PMC3406389

[b21] Chellappa S. L. . Acute exposure to evening blue-enriched light impacts on human sleep. J Sleep Res 22, 573–580 (2013). 23509952 10.1111/jsr.12050

[b22] Chang A. M., Aeschbach D., Duffy J. F. & Czeisler C. A. Evening use of light-emitting eReaders negatively affects sleep, circadian timing, and next-morning alertness. Proc Natl Acad Sci USA 112, 1232–1237 (2015). 25535358 10.1073/pnas.1418490112 PMC4313820

[b23] Arvanitogiannis A. & Amir S. Resetting the rat circadian clock by ultra-short light flashes. Neurosci Lett 261, 159–162 (1999). 10081973 10.1016/s0304-3940(99)00021-x

[b24] Nelson D. E. & Takahashi J. S. Sensitivity and integration in a visual pathway for circadian entrainment in the hamster (Mesocricetus auratus). J Physiol 439, 115–145 (1991). 1895235 10.1113/jphysiol.1991.sp018660 PMC1180102

[b25] Van Den Pol A. N., Cao V. & Heller H. C. Circadian system of mice integrates brief light stimuli. Am J Physiol 275, R654–657 (1998). 9688706 10.1152/ajpregu.1998.275.2.R654

[b26] Shigeyoshi Y. . Light-induced resetting of a mammalian circadian clock is associated with rapid induction of the mPer1 transcript. Cell 91, 1043–1053 (1997). 9428526 10.1016/s0092-8674(00)80494-8

[b27] Miyake S. . Phase-dependent responses of Per1 and Per2 genes to a light-stimulus in the suprachiasmatic nucleus of the rat. Neurosci Lett 294, 41–44 (2000). 11044582 10.1016/s0304-3940(00)01545-7

[b28] Butler M. P. & Silver R. Divergent photic thresholds in the non-image-forming visual system: entrainment, masking and pupillary light reflex. Proc Biol Sci 278, 745–750 (2011). 20861055 10.1098/rspb.2010.1509 PMC3030845

[b29] Shanahan T. L., Zeitzer J. M. & Czeisler C. A. Resetting the melatonin rhythm with light in humans. J Biol Rhythms 12, 556–567 (1997). 9406030 10.1177/074873049701200610

[b30] Glotzbach S. F., Edgar D. M. & Ariagno R. L. Biological rhythmicity in preterm infants prior to discharge from neonatal intensive care. Pediatrics 95, 231–237 (1995). 7838641

[b31] Mirmiran M., Maas Y. G. & Ariagno R. L. Development of fetal and neonatal sleep and circadian rhythms. Sleep Med Rev 7, 321–334 (2003). 14505599 10.1053/smrv.2002.0243

[b32] Maas C. . Relationship of salivary and plasma cortisol levels in preterm infants: results of a prospective observational study and systematic review of the literature. Neonatology 105, 312–318 (2014). 24603497 10.1159/000357555

[b33] Ivars K. . Development of Salivary Cortisol Circadian Rhythm and Reference Intervals in Full-Term Infants. PLoS One 10, e0129502 (2015). 26086734 10.1371/journal.pone.0129502 PMC4472813

[b34] Rahman S. A., Kollara A., Brown T. J. & Casper R. F. Selectively filtering short wavelengths attenuates the disruptive effects of nocturnal light on endocrine and molecular circadian phase markers in rats. Endocrinology 149, 6125–6135 (2008). 18687787 10.1210/en.2007-1742

[b35] Rivkees S. A. The Development of Circadian Rhythms: From Animals To Humans. Sleep Med Clin 2, 331–341 (2007). 19623268 10.1016/j.jsmc.2007.05.010 PMC2713064

